# Proteomics Challenges for the Assessment of Synuclein Proteoforms as Clinical Biomarkers in Parkinson’s Disease

**DOI:** 10.3389/fnagi.2022.818606

**Published:** 2022-03-31

**Authors:** Marie-Laure Pons, Neil Loftus, Jerome Vialaret, Stephane Moreau, Sylvain Lehmann, Christophe Hirtz

**Affiliations:** ^1^IRMB-PPC, INM, CHU Montpellier, INSERM, CNRS, Université de Montpellier, Montpellier, France; ^2^Shimadzu Corporation, Duisburg, Germany; ^3^Shimadzu Corporation, Manchester, United Kingdom

**Keywords:** proteoforms, Parkinson’s disease, Lewy bodies (LB), immunoassay, mass spectrometry

## Abstract

Parkinson’s disease is a complex neurodegenerative disorder resulting in a multifaceted clinical presentation which includes bradykinesia combined with either rest tremor, rigidity, or both, as well as many non-motor symptoms. The motor features of the disorder are associated with the pathological form of alpha synuclein aggregates and fibrils in Lewy bodies and loss of dopaminergic neurons in the substantia nigra. Parkinson’s disease is increasingly considered as a group of underlying disorders with unique genetic, biological, and molecular abnormalities that are likely to respond differentially to a given therapeutic approach. For this reason, it is clinically challenging to treat and at present, no therapy can slow down or arrest the progression of Parkinson’s disease. There is a clear unmet clinical need to develop reliable diagnostic and prognostic biomarkers. When disease-modifying treatments become available, prognostic biomarkers are required to support a definitive diagnosis and clinical intervention during the long prodromal period as no clinical implications or symptoms are observed. Robust diagnostic biomarkers would also be useful to monitor treatment response. Potential biomarkers for the sporadic form of Parkinson’s disease have mostly included synuclein species (monomer, oligomer, phosphorylated, Lewy Body enriched fraction and isoforms). In this review, we consider the analysis of synuclein and its proteoforms in biological samples using proteomics techniques (immunoassay and mass spectrometry) applied to neurodegenerative disease research.

## Introduction

Parkinson’s disease (PD) is the second most common neurodegenerative disorder after Alzheimer’s disease that affects around 1% of the world’s population over the age of 60 (Cova and Priori, 2018; Mazurskyy and Howitt, 2019; Mehra et al., 2019). PD, Dementia of Lewy body (DLB) and Multi system atrophy (MSA) collectively form a disease family called synucleinopathies. These pathologies are characterized by motor dysfunctions such as resting tremor, bradykinesia, rigidity and postural instability (Cova and Priori, 2018; Mehra et al., 2019; Porro et al., 2019) whereas non-motor symptoms include an impairment in learning and memory, constipation, anosmia, olfactory deficits, sleep disturbances, fatigue, sexual dysfunction, anxiety and depression. The clinical diagnosis of the synucleinopathies is primarily through the identification of cardinal motor dysfunction using a modified Hoehn and Yahr scale without evidence of histology or biomarker (Mazurskyy and Howitt, 2019). Motor dysfunction in PD is associated with a degeneration of the main dopaminergic pathway including the progressive loss of dopaminergic neurons in the substantia nigra pars compacta (SNc) (Schmid et al., 2013; Cova and Priori, 2018; Mehra et al., 2019; Porro et al., 2019). However, when these pathologies are presented in advanced stages of PD more than 50% of the dopaminergic neurons have been lost (Schmid et al., 2013; Cova and Priori, 2018) despite first non-motor features often appearing 10–15 years earlier. Consequently, there is an unmet clinical need to develop new prospective biomarkers to discriminate between synucleinopathies, detect their presence at an early stage and monitor the progression of the disease by correlating with the Unified Parkinson’s Disease Rating Scale (UPDRS) or Hoehn and Yahr scale (Schmid et al., 2013).

One of the most widely cited biomarker candidates for PD, DLB and MSA is the α-synuclein protein (α-syn), as the deposition of fibrillar aggregates of α-syn in the cytoplasm of selective populations of neurons (PD and DLB) and oligodendroglia (MSA) can form pathogenic inclusions called Lewy bodies (LB) (Mehra et al., 2019; Porro et al., 2019; Mou et al., 2020). This protein initializes its oligomerization and fibrilization by a change in its conformational state and adopts a beta sheet conformation which is a characteristic pattern for PD and DLB (Killinger et al., 2018; Kumar and Kumar, 2019; Mazurskyy and Howitt, 2019; Mehra et al., 2019; Vivoli Vega et al., 2019). This conformational change can be due to genetic factors such as mutations (Mehra et al., 2019; Mou et al., 2020), environmental factors as oxidative stress, aging or reception of inflammatory cell signal (Mazurskyy and Howitt, 2019; Porro et al., 2019). α-Syn aggregates or fibrils accumulate in the intraneuronal space, leading to toxicity and neuronal death in neurodegenerative diseases (Oeckl et al., 2018; Mazurskyy and Howitt, 2019; Mehra et al., 2019). It is mostly found phosphorylated on serine 129 (pS129) and has multiple ubiquitination forms in LB (Mehra et al., 2019).

Cerebrospinal fluid (CSF) α-syn species, which include total alpha synuclein (t-α-syn), oligomers alpha synuclein (o-α-syn), pS129 and/or truncated forms, have been shown to be promising candidate biomarkers for PD given their roles in pathogenesis. However, when measured separately the expression levels in PD patients show a low specificity and marginal diagnostic accuracy. The less well studies CSF beta synuclein (β-syn) and gamma synuclein (γ-syn) species are both elevated in Alzheimer’s Disease (AD) and Creutzfeldt Jacob Disease (CJD), but not PD. β-syn levels have been shown to increase in Parkinson’s disease dementia (PDD) and patients with DLB in comparison to PD patients and controls, but it is absent in LB which is the hallmark of synucleinopathies (Oeckl et al., 2016).

In this context, the synuclein family and their proteoforms are considered as potential biomarkers for neurodegenerative diseases (Oeckl et al., 2016, 2018, 2020). The immunoassay methodologies suggest there is a marginal reduction in CSF α-syn in PD patients, however, the variability of the technique has led to large concentration differences between studies and inconclusive results. The variability in immunoassay-based results may be attributed to the divergent antibodies that recognize different fragments of the protein with variable affinity. It may also reflect a need to standardize study design and methodologies. As an antibody-free platform, targeted mass spectrometry (MS) techniques such as multiple reaction monitoring (MRM) is routinely used in quantitative proteomics delivering high sensitivity, accuracy, and multiplex capability.

In this review, we consider the proteomics challenges for the assessment of synuclein proteoforms as clinical biomarkers in Parkinson’s disease using mass spectrometry as an alternative to antibody-based assays.

## Synuclein Structure, Function and Modification in Physiological Versus Pathological State

The synuclein family is composed of 3 main proteins namely α-syn, β-syn and γ-syn (Payton et al., 2001; Öhrfelt et al., 2011; Oeckl et al., 2016; Gámez-Valero and Beyer, 2018; Mehra et al., 2019). These proteins are pre-synaptic proteins that are implicated in neurodegenerative diseases (Jia et al., 1999; Payton et al., 2001; Mehra et al., 2019).

### Structure

Under physiological conditions, α-syn is a protein of 140 amino acids with a molecular mass of 14,460 Da. This protein is coded by the *SNCA* gene (Emamzadeh, 2016; Gámez-Valero and Beyer, 2018; Mehra et al., 2019) and mostly found as a soluble monomeric unfolded protein (Chanthamontri et al., 2009; Porro et al., 2019) or bound to the membrane in dopaminergic neurons under double helix alpha conformation (Gámez-Valero and Beyer, 2018; Paciotti et al., 2018; Mazurskyy and Howitt, 2019; Mehra et al., 2019; Ponzini et al., 2019). In a pathological condition, α-syn adopts a beta sheet conformation (Mazurskyy and Howitt, 2019; Mehra et al., 2019; Porro et al., 2019) believed to be the starting point for oligomerization and fibrillization (Emamzadeh, 2016; Paciotti et al., 2018; Mehra et al., 2019).

The α-syn protein structure consists of three regions; N-terminal, a central non-amyloid component (NAC) and a C-terminal (Chanthamontri et al., 2009; Ponzini et al., 2019). The N-terminal region (amino-acid residues 1–60) has an hexameric motif with a repeated sequence of 7 amino acids KTKEGV (Spillantini et al., 1995; Chanthamontri et al., 2009; Emamzadeh, 2016). This sequence forms an amphipathic α-helix enabling it to bind to membranes (Chanthamontri et al., 2009; Gámez-Valero and Beyer, 2018; Paciotti et al., 2018; Mehra et al., 2019). The central NAC region (61–95) is responsible for protein aggregation and protein-protein interaction (Chanthamontri et al., 2009; Emamzadeh, 2016; Gámez-Valero and Beyer, 2018; Mehra et al., 2019; Ponzini et al., 2019). Finally, the C-terminal region (96–140) has a higher amino acid content (Chanthamontri et al., 2009) and confers a negative charge to the protein even at physiological pH (Mazurskyy and Howitt, 2019; Ponzini et al., 2019). It is also highly composed of modifications such as truncations and post translational modifications (PTMs) playing an important role in the aggregation propensity of the protein (Chanthamontri et al., 2009; Emamzadeh, 2016; Gámez-Valero and Beyer, 2018; Mehra et al., 2019).

β-syn and γ-syn, are also found as unstructured monomer protein in a physiological state (Gámez-Valero and Beyer, 2018; Mehra et al., 2019). β-Syn is a 134 amino-acid residue protein with a molecular mass of 14,288 Da (Gámez-Valero and Beyer, 2018; Mehra et al., 2019) and shares structural similarities with α-syn. β-syn differs from α-syn in the deletion of 11 amino acids (amino-acid residues 73–83) in the NAC region which results in a lower propensity to form amyloid fibrils and a higher α-helical propensity. γ-Syn is a smaller isoform with 127 amino-acid residues and a molecular mass of 13,331 Da (Mehra et al., 2019).

### Function

The physiological function of α-syn is to regulate vesicular plasticity, oxidative stress, and mitochondrial function (Pratt et al., 2015). It is richly expressed in the central nervous system and is associated with regulating neurotransmitter release (Emamzadeh, 2016; Mazurskyy and Howitt, 2019; Mehra et al., 2019), synaptic plasticity (Emamzadeh, 2016; Mehra et al., 2019; Porro et al., 2019), vesicle trafficking (Emamzadeh, 2016; Mehra et al., 2019), brain lipid metabolism, remodeling membranes and formation of membrane channels (Gámez-Valero and Beyer, 2018; Paciotti et al., 2018). In a pathological state, the function of α-syn still remains elusive despite evidence of its neurotoxic role in synucleinopathies forming aggregates, fibrils and oligomers present in brain (Schmid et al., 2013; Oeckl et al., 2018). Pathological α-syn is known as prion-like or prionoid, given that abnormal α-syn can spread to neighboring brain regions and cause aggregation of endogenous α-syn in these regions like seeds, in a “prion-like” manner. This “prion-like” hypothesis considers common pathological properties between pathological α-syn and prions; seeding/templating, spreading from one cell to another, generating structurally differentiated conformations, and causing neurodegeneration. Recent studies now consider the gastrointestinal tract, including the appendix as the starting point for pathological α-syn aggregation. Once formed, the α-syn aggregates propagate from gut to brain *via* the vagus nerve. Pathological degeneration in the brain is thought to occur by self-aggregation and transmission infecting nearby naïve cells by endocytosis, exosome or creating tunneling nanotubes in the membrane (Mazurskyy and Howitt, 2019).

β-Syn is also widely expressed in the central nervous system but its impact on human brain disorders is currently enigmatic. It appears to have a motor function in neuronal systems (Gámez-Valero and Beyer, 2018) and could be a marker of the synapse degeneration due to its high level found in CSF and serum of AD and CJD patients (Oeckl et al., 2016, 2020). β-syn is also thought to play a role in the propensity of aggregation of α-syn in neurodegenerative diseases as β-syn has been shown to inhibit α-syn aggregation (Oeckl et al., 2016, 2020).

γ-Syn is also known to generate aggregates and polymorphisms in the γ-syn locus and has been associated with human diffuse LBD. As abnormal γ-syn is accumulated in neurons associated with neurodegenerative disease it is suggested that γ-syn aggregates play an important, but poorly understood, role in brain disorders (Oeckl et al., 2016).

### Synucleins Proteoforms Description

Synucleinopathies share a common pathogenic mechanism involving α-syn aggregation and progressive deposition of misfolded proteins; however, this mechanism is far from simple and the role of diverse PTMs, aggregate formations, and truncations, all of which contribute to a growing known set of proteoforms. Proteoforms describe all protein variants of a single gene including PTMs and sequence variants (Smith and Kelleher, 2013; Aebersold et al., 2018). In this section, we will review the published research into synuclein proteoforms on biofluid or brain tissue of patients with synucleinopathies.

### Mutations

α-Syn is a widely expressed protein encoded by the *SNCA* gene. *SNCA* was first identified as a causative agent in autosomal dominant PD and currently there are seven missense mutations (A30P, E46K, H50Q, G51D, A53T, A53E, and A53V) reported to be associated with familial PD (Payton et al., 2001; Chanthamontri et al., 2009; Muntané et al., 2012; Marotta et al., 2015; Flagmeier et al., 2016; Gámez-Valero and Beyer, 2018; Mehra et al., 2019). Each mutation is the result of a single point substitution in its nucleotide sequence. The first published mutation identified A53T substitution in α-syn resulting from an autosomal-dominant single base pair change in *SNCA*. Following this discovery, further autosomal dominant mutations in the *SNCA* gene have been found to cause familial PD including E46K, H50Q, G51D, A53T, A53E, A53V and A30P (Flagmeier et al., 2016). This suggests that the mutations in the N-ter part of the protein are not only responsible for the double helix alpha conformation of the protein but also the process of accelerating aggregation (or fibrilization) (Flagmeier et al., 2016). To our knowledge no such mutations have been reported for beta or gamma synuclein in synucleinopathies.

### Alternative Splicing

**FIGURE 1:**
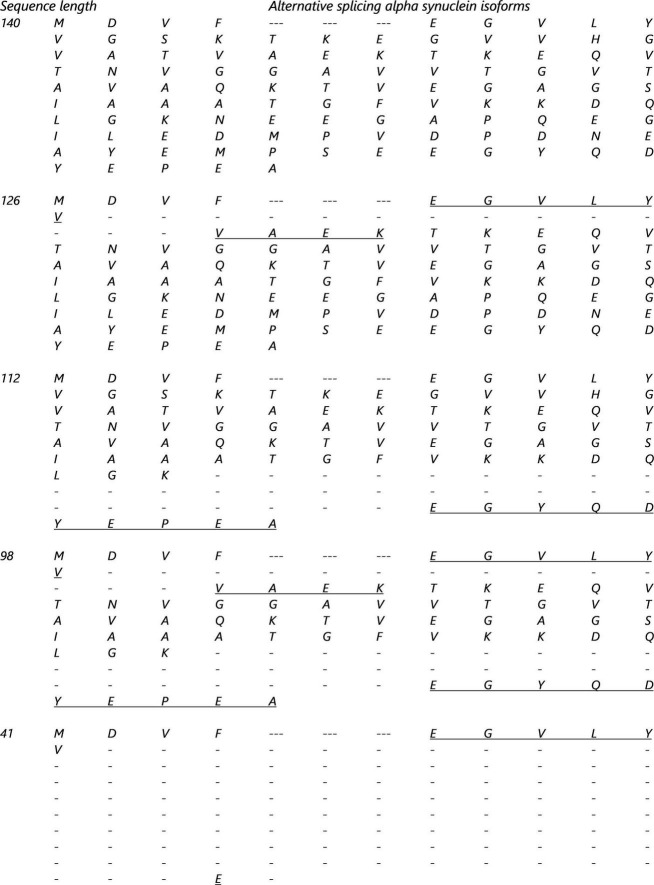
Alternative splicing isoforms sequences. The underline amino acids are proteotypic and tryptic peptides created by the alternative splicing of the SNCA gene: Common part of all the isoforms and are composed of the following residue [MKGLSKAKEGVVAAAEKTKQGVAEAAGKTK].

The synuclein family is encoded by three specific genes (Oeckl et al., 2016; Mehra et al., 2019), the *SNCA* gene codes for α-syn, *SNCB* gene codes for β-syn and *SNCG* codes for γ-syn. The most common isoform of the α-syn is 140 amino acids long and is also known as full-length α-syn. But due to alternative splicing of the *SNCA* gene mRNA, 4 other isoforms have been reported and described in Figure 1. In total 6 exons constitute the α-syn sequence but only 5 exons code the sequence. For example, the α-syn-126 lacks the amino sequence from 41 to 54 (exon 3) (Öhrfelt et al., 2011), α-syn-112 has a missing sequence between amino acids 103–130 (exon 5). The α-syn isoforms 98 and 41 skip two exons each, exon 3 and 5 (McLean et al., 2012; Öhrfelt et al., 2011; Vinnakota et al., 2018) and exon 3 and 4 (Vinnakota et al., 2018), respectively.

All isoforms (α-syn-140, α-syn-126, α-syn-112, α-syn-98 and α-syn-41) have been found in PD patients with synucleinopathies and are expressed differentially compared to controls (Vinnakota et al., 2018). In patients with PD, both wild type α-syn and isoform α-syn-126 levels were increased. Conversely, in DLB, it’s the isoform α-syn-112 that was increased and suggests that the α-syn isoforms play a pathological role (Vinnakota et al., 2018) and they may be potential biomarkers to distinguish between synucleinopathies (and AD).

β-Synuclein is encoded by the *SNCB* gene and has the same *SNCA*-like transcripts lacking either exon 3 or 5 (Beyer et al., 2011). Exon 3 lacking transcripts results in β-syn-120 and exon 5 lacking transcripts produce β-syn-104 (Jakes et al., 1994; Spillantini et al., 1995; Jensen et al., 1997; Lavedan et al., 1998a,b; Beyer et al., 2012; Gámez-Valero et al., 2018; Jain et al., 2018; Van de Vondel et al., 2018; Lodygin et al., 2019).

Both α-syn and β-syn genes *SNCA* and *SNCB* have very similar homology and undergo similar splicing events that create a trigger for the development of DLB and MSA and seems likely to affect other disease groups.

The γ-syn gene *SNCG* has only one isoform and has been reported to be missing one of the 5 coding exons due to alternative splicing (George, 2002).

### Post-translational Modifications of α-Synuclein

**FIGURE 2:**
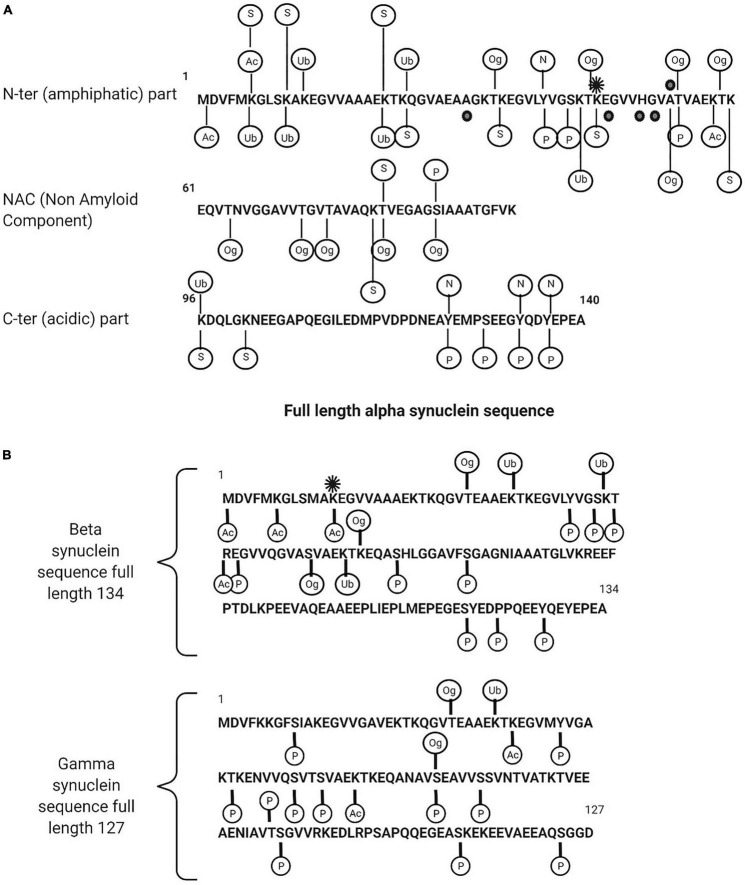
**(A)** Description of alpha synuclein post translational modifications. **(B)** Beta and gamma synuclein post translational modifications. Ac, acetylated; Ub, ubiquitination; Og, O-glyNaction; N, nitration; P, phosphorylated; S, SUMOYLATION. The black points are the point of mutations (in familial form of the PD) and the black star is methylation (mono).

Post-translational modifications (PTMs) for synucleins, particularly for α-syn, are extensive and includes phosphorylation, ubiquitination, nitration, truncation, and *O*-GlcNAcylation. All synuclein PTMs found in a physiological or pathological form in neurodegenerative diseases are described in Figures 2A,B and Tables 1, 2.

**TABLE 1:** Alpha synuclein truncation products (-AA): Nter truncation.

Truncation position	Truncation products
18	(−)AEKTKQGVAEAAGKTKEGVLYVGSKTKEGVVHGVATVAEKTKEQVTNVGGAVVTGVTAVAQKTVEGAGSIAAATGFVKKDQLGKNEEGAPQE GILEDMPVDPDNEAYEMPSEEGYQDYEPEA
19	(−)EKTKQGVAEAAGKTKEGVLYVGSKTKEGVVHGVATVAEKTKEQVTNVGGAVVTGVTAVAQKTVEGAGSIAAATGFVKKDQLGKNEEGAPQEGI LEDMPVDPDNEAYEMPSEEGYQDYEPEA
47	(−)VVHGVATVAEKTKEQVTNVGGAVVTGVTAVAQKTVEGAGSIAAATGFVKKDQLGKNEEGAPQEGILEDMPVDPDNEAYEMPSEEGYQDYEPEA
57	(−)KTKEQVTNVGGAVVTGVTAVAQKTVEGAGSIAAATGFVKKDQLGKNEEGAPQEGILEDMPVDPDNEAYEMPSEEGYQDYEPEA
63	(−)TNVGGAVVTGVTAVAQKTVEGAGSIAAATGFVKKDQLGKNEEGAPQEGILEDMPVDPDNEAYEMPSEEGYQDYEPEA
64	(−)NVGGAVVTGVTAVAQKTVEGAGSIAAATGFVKKDQLGKNEEGAPQEGILEDMPVDPDNEAYEMPSEEGYQDYEPEA
67	(−)GAVVTGVTAVAQKTVEGAGSIAAATGFVKKDQLGKNEEGAPQEGILEDMPVDPDNEAYEMPSEEGYQDYEPEA
70	(−)VTGVTAVAQKTVEGAGSIAAATGFVKKDQLGKNEEGAPQEGILEDMPVDPDNEAYEMPSEEGYQDYEPEA
71	(−)TGVTAVAQKTVEGAGSIAAATGFVKKDQLGKNEEGAPQEGILEDMPVDPDNEAYEMPSEEGYQDYEPEA
72	(−)GVTAVAQKTVEGAGSIAAATGFVKKDQLGKNEEGAPQEGILEDMPVDPDNEAYEMPSEEGYQDYEPEA*
75	(−)AVAQKTVEGAGSIAAATGFVKKDQLGKNEEGAPQEGILEDMPVDPDNEAYEMPSEEGYQDYEPEA*
79	(−)KTVEGAGSIAAATGFVKKDQLGKNEEGAPQEGILEDMPVDPDNEAYEMPSEEGYQDYEPEA
83	(−)GAGSIAAATGFVKKDQLGKNEEGAPQEGILEDMPVDPDNEAYEMPSEEGYQDYEPEA
84	(−)AGSIAAATGFVKKDQLGKNEEGAPQEGILEDMPVDPDNEAYEMPSEEGYQDYEPEA*
86	(−)SIAAATGFVKKDQLGKNEEGAPQEGILEDMPVDPDNEAYEMPSEEGYQDYEPEA
87	(−)IAAATGFVKKDQLGKNEEGAPQEGILEDMPVDPDNEAYEMPSEEGYQDYEPEA*

MDVFMKGLSKAKEGVVAAAEKTKQGVAEAAGKTKEGVLYVGSKTKEGVVHGVATVAEKTKEQVTNVGGAVVTGVTAVAQKTVEGAGSIAAATGFVKKDQLGKNEEGAPQEGILEDMPVDPDNEAYEMPSEEGYQDYEPEA. Main alpha synuclein isoform (α-syn-140) sequence. (ac) acetylated, (P) phosphorylated and * observed in blood cells (the others are described in PD brains).

**TABLE 2:** Alpha synuclein truncation products (AA-): Cter truncation.

Truncation position	Generated fragment
53	*(ac)MDVFMKGLSKAKEGVVAAAEKTKQGVAEAAGKTKEGVLYVGSKTKEGVVHGVA(−)
55	MDVFMKGLSKAKEGVVAAAEKTKQGVAEAAGKTKEGVLYVGSKTKEGVVHGVATV(−)
57	*(ac)MDVFMKGLSKAKEGVVAAAEKTKQGVAEAAGKTKEGVLYVGSKTKEGVVHGVATVAE(−)
71	*(ac)MDVFMKGLSKAKEGVVAAAEKTKQGVAEAAGKTKEGVLYVGSKTKEGVVHGVATVAEKTKEQVTNVGGAVV(−)
73	MDVFMKGLSKAKEGVVAAAEKTKQGVAEAAGKTKEGVLYVGSKTKEGVVHGVATVAEKTKEQVTNVGGAVVTG(−)
74	*(ac)MDVFMKGLSKAKEGVVAAAEKTKQGVAEAAGKTKEGVLYVGSKTKEGVVHGVATVAEKTKEQVTNVGGAVVTGV(−)
83	*(ac)MDVFMKGLSKAKEGVVAAAEKTKQGVAEAAGKTKEGVLYVGSKTKEGVVHGVATVAEKTKEQVTNVGGAVVTGVTAVAQKTVE(−)
91	*(ac)MDVFMKGLSKAKEGVVAAAEKTKQGVAEAAGKTKEGVLYVGSKTKEGVVHGVATVAEKTKEQVTNVGGAVVTGVTAVAQKTVEGAGSIAAA(−)
94	MDVFMKGLSKAKEGVVAAAEKTKQGVAEAAGKTKEGVLYVGSKTKEGVVHGVATVAEKTKEQVTNVGGAVVTGVTAVAQKTVEGAGSIAAATGF(−)
96	MDVFMKGLSKAKEGVVAAAEKTKQGVAEAAGKTKEGVLYVGSKTKEGVVHGVATVAEKTKEQVTNVGGAVVTGVTAVAQKTVEGAGSIAAATG FVK(−)
97	MDVFMKGLSKAKEGVVAAAEKTKQGVAEAAGKTKEGVLYVGSKTKEGVVHGVATVAEKTKEQVTNVGGAVVTGVTAVAQKTVEGAGSIAAATGF VKK(−)
101	MDVFMKGLSKAKEGVVAAAEKTKQGVAEAAGKTKEGVLYVGSKTKEGVVHGVATVAEKTKEQVTNVGGAVVTGVTAVAQKTVEGAGSIAAATGF VKKDQLG(−)
103	MDVFMKGLSKAKEGVVAAAEKTKQGVAEAAGKTKEGVLYVGSKTKEGVVHGVATVAEKTKEQVTNVGGAVVTGVTAVAQKTVEGAGSIAAATGF VKKDQLGKN(−)
104	MDVFMKGLSKAKEGVVAAAEKTKQGVAEAAGKTKEGVLYVGSKTKEGVVHGVATVAEKTKEQVTNVGGAVVTGVTAVAQKTVEGAGSIAAATGF VKKDQLGKNE(−)
113	MDVFMKGLSKAKEGVVAAAEKTKQGVAEAAGKTKEGVLYVGSKTKEGVVHGVATVAEKTKEQVTNVGGAVVTGVTAVAQKTVEGAGSIAAATGF VKKDQLGKNEEGAPQEGIL(−)
114	MDVFMKGLSKAKEGVVAAAEKTKQGVAEAAGKTKEGVLYVGSKTKEGVVHGVATVAEKTKEQVTNVGGAVVTGVTAVAQKTVEGAGSIAAATGF VKKDQLGKNEEGAPQEGILE(−)
115	MDVFMKGLSKAKEGVVAAAEKTKQGVAEAAGKTKEGVLYVGSKTKEGVVHGVATVAEKTKEQVTNVGGAVVTGVTAVAQKTVEGAGSIAAATGF VKKDQLGKNEEGAPQEGILED(−)
116	MDVFMKGLSKAKEGVVAAAEKTKQGVAEAAGKTKEGVLYVGSKTKEGVVHGVATVAEKTKEQVTNVGGAVVTGVTAVAQKTVEGAGSIAAATGF VKKDQLGKNEEGAPQEGILEDM(−)
117	MDVFMKGLSKAKEGVVAAAEKTKQGVAEAAGKTKEGVLYVGSKTKEGVVHGVATVAEKTKEQVTNVGGAVVTGVTAVAQKTVEGAGSIAAATGF VKKDQLGKNEEGAPQEGILEDMP(−)
119	MDVFMKGLSKAKEGVVAAAEKTKQGVAEAAGKTKEGVLYVGSKTKEGVVHGVATVAEKTKEQVTNVGGAVVTGVTAVAQKTVEGAGSIAAATGF VKKDQLGKNEEGAPQEGILEDMPVD(−)
120	MDVFMKGLSKAKEGVVAAAEKTKQGVAEAAGKTKEGVLYVGSKTKEGVVHGVATVAEKTKEQVTNVGGAVVTGVTAVAQKTVEGAGSIAAATGF VKKDQLGKNEEGAPQEGILEDMPVDP(−)
121	MDVFMKGLSKAKEGVVAAAEKTKQGVAEAAGKTKEGVLYVGSKTKEGVVHGVATVAEKTKEQVTNVGGAVVTGVTAVAQKTVEGAGSIAAATGF VKKDQLGKNEEGAPQEGILEDMPVDPD(−)
122	MDVFMKGLSKAKEGVVAAAEKTKQGVAEAAGKTKEGVLYVGSKTKEGVVHGVATVAEKTKEQVTNVGGAVVTGVTAVAQKTVEGAGSIAAATGF VKKDQLGKNEEGAPQEGILEDMPVDPDN(−)
123	MDVFMKGLSKAKEGVVAAAEKTKQGVAEAAGKTKEGVLYVGSKTKEGVVHGVATVAEKTKEQVTNVGGAVVTGVTAVAQKTVEGAGSIAAATGF VKKDQLGKNEEGAPQEGILEDMPVDPDNE(−)
124	MDVFMKGLSKAKEGVVAAAEKTKQGVAEAAGKTKEGVLYVGSKTKEGVVHGVATVAEKTKEQVTNVGGAVVTGVTAVAQKTVEGAGSIAAATGF VKKDQLGKNEEGAPQEGILEDMPVDPDNEA(−)
125	MDVFMKGLSKAKEGVVAAAEKTKQGVAEAAGKTKEGVLYVGSKTKEGVVHGVATVAEKTKEQVTNVGGAVVTGVTAVAQKTVEGAGSIAAATGF VKKDQLGKNEEGAPQEGILEDMPVDPDNEAY(−)
126	MDVFMKGLSKAKEGVVAAAEKTKQGVAEAAGKTKEGVLYVGSKTKEGVVHGVATVAEKTKEQVTNVGGAVVTGVTAVAQKTVEGAGSIAAATGF VKKDQLGKNEEGAPQEGILEDMPVDPDNEAYE(−)
129	MDVFMKGLSKAKEGVVAAAEKTKQGVAEAAGKTKEGVLYVGSKTKEGVVHGVATVAEKTKEQVTNVGGAVVTGVTAVAQKTVEGAGSIAAATGF VKKDQLGKNEEGAPQEGILEDMPVDPDNEAYEMPS(−)
133	*(ac)MDVFMKGLSKAKEGVVAAAEKTKQGVAEAAGKTKEGVLYVGSKTKEGVVHGVATVAEKTKEQVTNVGGAVVTGVTAVAQKTVEGAGSIA AATGFVKKDQLGKNEEGAPQEGILEDMPVDPDNEAYEMPSEEGY(−)
133	MDVFMKGLSKAKEGVVAAAEKTKQGVAEAAGKTKEGVLYVGSKTKEGVVHGVATVAEKTKEQVTNVGGAVVTGVTAVAQKTVEGAGSIAAATGF VKKDQLGKNEEGAPQEGILEDMPVDPDNEAYEMPSEEGY(−)
135	MDVFMKGLSKAKEGVVAAAEKTKQGVAEAAGKTKEGVLYVGSKTKEGVVHGVATVAEKTKEQVTNVGGAVVTGVTAVAQKTVEGAGSIAAATGF VKKDQLGKNEEGAPQEGILEDMPVDPDNEAYEMPSEEGYQD(−)
137	*(ac)MDVFMKGLSKAKEGVVAAAEKTKQGVAEAAGKTKEGVLYVGSKTKEGVVHGVATVAEKTKEQVTNVGGAVVTGVTAVAQKTVEGAGSIA AATGFVKKDQLGKNEEGAPQEGILEDMPVDPDNEAYEMPS(P)EEGYQDYE(−)
139	*(ac)MDVFMKGLSKAKEGVVAAAEKTKQGVAEAAGKTKEGVLYVGSKTKEGVVHGVATVAEKTKEQVTNVGGAVVTGVTAVAQKTVEGAGSIA AATGFVKKDQLGKNEEGAPQEGILEDMPVDPDNEAYEMPS(P)EEGYQDYEPE(−)

(ac) acetylated, (P) phosphorylated and * observed in blood cells (the others are described in PD brains).

A common PTM of α-syn is phosphorylation on serine 129 (pS129-α-syn) (Okochi et al., 2000; Pronin et al., 2000; Fujiwara et al., 2002; Anderson et al., 2006; Inglis et al., 2009; Sakamoto et al., 2009; Mbefo et al., 2010; Schmid et al., 2013; Kellie et al., 2014; Khalaf et al., 2014; Pratt et al., 2015). It is markedly upregulated in PD and related synucleinopathies with more than 90% found in LB compared to less than 5% in unaffected brains, suggesting that phosphorylation is a key determinant in the rate of aggregation and toxicity. The quantitation of total α-syn including its oligomeric and phosphorylated forms has been measured in human CSF and plasma by a range of analytical techniques and the findings are consistent in reporting higher levels of pS129 in PD patients compared to controls (Chalatsa et al., 2020). α-Syn is also phosphorylated on a number of other residues including Y39 (Burmann et al., 2020), S42 (Stuart et al., 2015) in the N terminus, Y125, Y133, and Y136 in the C terminus, and serine 87 (pS87-α-syn) in the NAC region. pS87-α-syn is of interest as it is one of the few sites that differentiates human α-syn from rat and mouse and it is considered as a key site of α-syn aggregation and fibrillogenesis (Paleologou et al., 2010).

Although pS129-α-syn is the dominant PTM found in LB, further PTM’s are also found which include ubiquitination at Lys residues 12, 21, 23, 43 and 96 and specific Cter truncations at Asp-115, Asp-119, Asn-122, Tyr-133, and Asp-135 (Anderson et al., 2006). Both phosphorylation and ubiquitination have emerged as consistent markers of α-syn pathology. pS129-α-syn and ubiquitinated α-syn at multiple sites (the major α-synuclein species in LBs is mono-, di-, and tri-ubiquitinated) have been detected in the CSF and plasma from PD, MSA, LB dementia cases and from controls. The extent to which the concentration in CSF reflects the disease progression or severity is not known or easily interpretated (Schmid et al., 2013).

Conversely, *O*-GlcNAcylated α-syn PTM appears to reduce neurotoxicity by inhibiting the aggregation of aggressive mutants of α-synuclein. *O*-GlcNAcylated is an extensive α-syn PTM of serine and threonine residues. α-Syn is *O*-GlcNAcylated at eight different threonine residues 33, 44, 53, 54 (Steentoft et al., 2013), 59, 64, 72 (Levine et al., 2019), 75 and 81 (Levine et al., 2017) and a single serine residue 87. Using synthetic protein chemistry, studies have shown site specific inhibitory effects on aggregation and cellular toxicity.

Despite the dominance of phosphorylated α-syn in LB’s with 90% or more found as pS129-α-syn, 15–20% are present as C-terminally truncated α-syn. Major C-ter truncations are common in synucleinopathies, including those cleaved after residues 103, 115, 119, 122, 125, 133, and 135 (Sorrentino and Giasson, 2020) but are not common in healthy controls. Other truncations have been reported in hematopoietic cells from healthy controls, for example, Nter truncation fragment 72–140, 75–140, 84–140, 87–140 and acetylated Cter truncation 1–53, 1–57, 1–71, 1–74, 1–83, 1–91, 1–133 and 1–137. Fragments 10–57 and 54–83 are combined Nter and Cter truncations (Melani et al., 2022). Inhibiting truncation of α-syn has been shown to prevent fibrillation and aggregation, suggesting it may be a potential therapeutic target.

β-syn and γ-syn are also subjected to extensive PTM including phosphorylation, ubiquitination, nitration, truncation, and *O*-GlcNAcylation (Inglis et al., 2009; Viodé, 2018; Mollenhauer et al., 2019; Viodé et al., 2019), however, few studies are reported in literature and the impact of β-syn and γ-syn PTM’s on the pathology of neurodegenerative disease is poorly understood. β-syn and γ-syn PTMs are illustrated in Figure 2B.

All these specific forms (mutations, isoforms, PTMs and protein family) have been proposed as putative biomarkers for synucleinopathies as their detection is specifically associated with patient PD disease (or in insoluble fraction as LBs) or are observed in different levels of expression compared to controls in biological samples.

## Clinical Immunoassay in Biofluids

Clinical proteomics has been dominated by antibody-based detection techniques (Schmid et al., 2013). It is simple to integrate into routine laboratories, highly automated, sensitive, and reproducible. It has been successfully applied to a diverse range of biomarker discovery diagnostics including AD by measuring total tau, phospho-tau and Abeta40/Abeta42 ratio using immunoassay into patient CSF (Parnetti and Eusebi, 2018). However, such immunoassay methods have recognized limitations for synuclein analysis given the difference in absolute concentrations between commercially available assays for the analysis of “total” α-synuclein in CSF (Mollenhauer et al., 2019).

In this context, mass spectrometry could play an important role in the research field of neurodegenerative diseases due to its robustness, multiplexing, specificity, and lack of dependence on available AB (Oeckl et al., 2018; Paciotti et al., 2018).

## Mass Spectrometry Identification and Quantification of Synuclein

Recent advances in mass spectrometry (MS) based clinical proteomics are the result of new developments not only in MS detection and its capability to measure with high specificity and sensitivity but also with advances in sample preparation, peptide separation, data analysis and the ability to multiplex proteins and discover PTMs in a single assay (Chanthamontri et al., 2009; Viodé, 2018; Viodé et al., 2019). MS-based proteomics methods use two principal approaches: bottom-up and top-down proteomics.

Bottom-up proteomics methods typically use individual proteases or protease mixtures to selectively cleave proteins at multiple amino acid sites to produce a mixture of small peptides. Protein digestion creates small peptide fragments which are well suited to MS detection, limited number of charges on each peptide, increased LC separation efficiency, and increased sample homogeneity. It is the most widely used approach for protein quantitation, identification, and characterization. However, there are limitations as the sequence coverage information may not fully describe the location and number of PTMs and endogenous proteolysis may be incomplete. The main protease used in the bottom-up approach is trypsin an enzyme that cleaves proteins at the carboxyterminal of Arg and Lys residues, resulting in a positive charge at the peptide C-terminus. All the synuclein generated tryptic peptides are shown in Table 3.

**TABLE 3:** Synuclein generated tryptic peptides (observed or not in literature).

Protein name	Generated tryptic peptides	Position on the sequence	Proteotypic Peptide ?	Already observed ?
α-syn	MDVFMK or (ac)MDVFMK	1–6	Common with β-syn	Yes, both form
	EGVVAAAEK	13–21	Common with β-syn	Yes
	QGVAEAAGK	24–32	Proteotypic	Yes
	EGVLYVGSK	35–43	Common with β-syn	Yes
	EGVVHGVATVAEK	46–58	Proteotypic	Yes
	EQVTNVGGAVVTGVTAVAQK	61–80	Proteotypic	Yes
	TVEGAGSIAAATGFVK	81–96	Proteotypic	Yes
	DQLGK	98–102	Proteotypic	Yes
	NEEGAPQEGILEDMPVDPDNEAYEMPSEEGYQDYEPEA	103–140	Proteotypic	No
β-syn	QGVTEAAEK	24–32	Common with γ-syn	No
	EGVVQGVASVAEK	46–58	Proteotypic	Yes
	EQASHLGGAVFSGAGNIAAATGLVK	61–85	Proteotypic	Yes
	REEFPTDLKPEEVAQEAAEEPLIEPLMEPEGESYEDPPQEEYQEYEPEA	86–134	Proteotypic	No
γ-syn	EGVVGAVEK	13–21	Proteotypic	No
	EGVMYVGAK	35–43	Proteotypic	No
	ENVVQSVTSVAEK	46–58	Proteotypic	Yes
	EQANAVSEAVVSSVNTVATK	61–80	Proteotypic	No
	TVEEAENIAVTSGVVR	81–96	Proteotypic	Yes
	EDLRPSAPQQEGEASK	98–113	Proteotypic	No
	EEVAEEAQSGGD	116–127	Proteotypic	No

**FIGURE 3:**
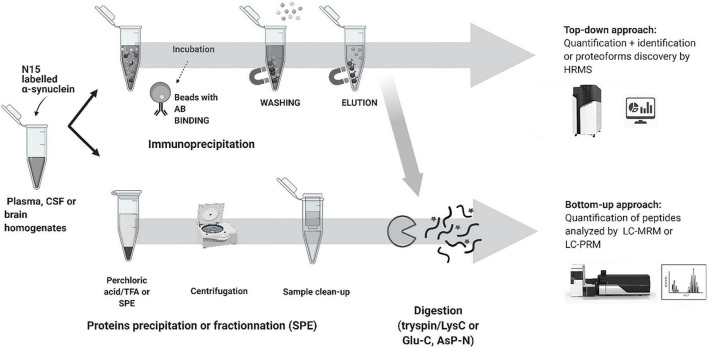
Typical workflow used for synuclein analysis by LC-MS.

Top-down proteomics analyzes intact proteins and proteoforms that have not been subject to proteolytic digestion. Top-down approaches provide more information to identify and quantify distinct proteoforms that would have been lost by endoproteinase digestion. The principal limitation of the top-down approach relates to detecting high molecular weight proteins > 50 kDa and low abundance proteoforms with charge envelope broadening being particularly difficult to detect with LC co-elution of a high abundant component. Both workflows and approaches are shown in Figure 3 for α-syn analysis.

To consider the best-practice in developing MS based methods for clinical proteomics including α-syn analysis, this section will review critical aspects of the workflow.

### Standards for Quantitation

Most quantitative proteomics analyses use isotopic labeling of proteins or peptides as internal standards, which can then be differentiated by MS and used to correct for analytical variability within the assay. There are several approaches: relative, absolute quantitation, and label-free strategies.

Relative quantitation methods include labeling techniques such as Stable Isotope Labeling by Amino acids in Cell culture (SILAC), Isotope-Coded Affinity Tag, (ICAT), Isotope-Coded Protein Labeling (ICPL) and isobaric tags comparing whole proteomes or the relative amount of a high number of proteins. Absolute quantitation determines the concentration of distinct proteins within a sample.

A review of label-based internal standardization applied to α-syn in CSF is shown below (Oeckl et al., 2018).

• PSAQ: Protein standard absolute quantification which targets 15N labeled human recombinant protein. This approach takes into account analytical variance in sample preparation, matrix effects and instrument performance.
• WISIL: Winged Stable isotope labeled (SIL) peptides (also termed extended, flanked, or cleavable SIL peptides), involves a sequence of the peptide being extended by amino acids labeled to control digestion and could therefore be added before the digestion step.
• SIL: corresponds to the exact sequence of peptides generated with heavy labeling. Consequently, this standard is added after the digestion step.
• QrPREST: Quantitative protein epitope signature tags with longer sequences, between 50 and 150 residues length, with heavy labeling. This approach results in a longer protein sequence which may better reflect the effects of sample preparation on the native protein and deliver higher quantitative precision.

15N labeled human recombinant protein has been used to quantify α-syn (Oeckl et al., 2018; Bhattacharjee et al., 2019; Singh et al., 2020) and β-syn (Oeckl et al., 2016) in brain tissue and CSF samples by LC-MS/MS with MRM. Heavy labeled peptides, 13C and 15N labeled C-ter lysine, has been applied to the analysis of α-syn (Oeckl et al., 2016, 2018; Bhattacharjee et al., 2019; Viodé et al., 2019), β-syn and γ-syn (Oeckl et al., 2016). The efficacity of each label-based internal standard approach is highly dependent on the ability to correct for analytical variability at different steps of the method (fractionation/purification, digestion, LC-MS/MS analysis) and each approach needs to be carefully considered to optimize the accuracy and precision of the assay.

### Enzyme Used for Synuclein Digestion

Bottom-up quantitative proteomics is dependent on a protease to digest proteins into peptides, which are then identified and quantified by LC-MS/MS. The most commonly used protease is trypsin, due to its widespread availability and ease of use. However, proteolytic digestion by trypsin alone can be a limiting factor in peptide-centric proteomics particularly when working with aggregation-prone proteins and amyloid-forming proteins such as α-syn. α-Syn trypsin proteolysis generates a large, hydrophilic, acidic C-terminal fragment of residues 103–140 carrying 11 glutamate or aspartate residues and 2 methionine. It is inherently complex due to the heterogeneity of the PTMs that can occur within this C-terminal region.

An alternative proteolytic approach using GluC (Mollenhauer et al., 2019; Viodé et al., 2019) has been shown to access the α-syn C-terminal region with greater specificity to identify all pathologically relevant PTMs (namely pY125 or pS129, nY133, and other nitrated C-terminal Tyr residues). A combined proteolytic approach with trypsin/ASP-N (Yang et al., 2017), trypsin/Gluc (Mollenhauer et al., 2019) and trypsin/lysC have been used to increase sequence coverage and identify signature peptides with a high specificity (Guerreiro et al., 2012; Oeckl et al., 2016, 2018; Yang et al., 2017; Bhattacharjee et al., 2019; Singh et al., 2020).

### Matrix Effect and Sample Preparation

In quantitative proteomics, the detection of low abundance proteins such as α-syn remains a considerable challenge due to the high dynamic range of the human proteome and the impact of ion suppression in the MS. Front-end protein enrichment strategies are critical in any workflow to capture and enrich low-abundance proteins from the complex biological sample before LC-MS/MS analysis. Several methods have been used to negate highly abundant proteins in α-syn analysis including protein precipitation (Yang et al., 2017; Viodé et al., 2019; Singh et al., 2020), immunoprecipitation (IP) (Guerreiro et al., 2012; Kellie et al., 2014; Killinger et al., 2018; Bhattacharjee et al., 2019; Mollenhauer et al., 2019; Oeckl et al., 2020) direct digestion (Oeckl et al., 2016, 2018) and SDS-gel (Guerreiro et al., 2012; Lamontagne-Proulx et al., 2019), mostly followed by clean-up. Protein precipitation (Yang et al., 2017; Viodé et al., 2019; Singh et al., 2020) and IP (Guerreiro et al., 2012; Kellie et al., 2014; Killinger et al., 2018; Bhattacharjee et al., 2019; Mollenhauer et al., 2019; Oeckl et al., 2020), are commonly cited for the analysis of α-syn in biofluids.

### Cohort

In all clinical studies, PD patient cohorts and control groups must be carefully considered to record clinically relevant information in a standardized manner that can be performed at regular intervals. This information should include Hoehn and Yahr scale, Unified Parkinson’s Disease Rating Scale, Braak staging in addition to clinical predictors of group assignment include sex, age at disease onset, presence of tremor as a predominant clinical feature, years of education, and cognitive impairment at onset. Other detailed information on comorbidities, head injury, complications of dopaminergic therapy, autonomic dysfunction (orthostatic symptoms, anhidrosis/hyperhidrosis, urinary incontinence), sleep disorders, dysphagia, anxiety, and depression have been used in to help identify predictors of disease progression (Sorrentino and Giasson, 2020). A clinical evaluation according to international diagnostic criteria should also be confirmed by neuropathologic examination. Studies should also consider a 3 year follow up to understand the rate of disease progression, identify possible new red flags and test for levodopa responsiveness.

### Sample Collection, Storage and Sample Pre-treatment

Patient sample collection must conform to governmental and institutional guidelines and consider protocols that will optimize the quantitation and identification of synucleins in biological samples. This includes sample storage temperature, storage time, time before processing (from sample to centrifuge), sample mixing (adding an anti-coagulant), checking for blood contamination, sample storage and delivery (with ice or room temperature, in boxes or not) (Mollenhauer et al., 2017; Markopoulou et al., 2020).

### Biological Sample

Pre-analytical, analytical and assay-related issues influence interindividual variability and interstudy variability of α-syn levels and require careful consideration. Biomarker assays developed for CSF, saliva or urine requires a check for blood contamination and samples with > 50/μL or hemoglobin level > 200 ng/mL should be excluded (evidence level) (Mollenhauer et al., 2019). Blood contamination in CSF lumbar puncture can markedly affect the reported concentration of α-syn and γ-syn levels in CSF (not for β-syn) (Guerreiro et al., 2012; Kang et al., 2016; Oeckl et al., 2016, 2018; Yang et al., 2017; Killinger et al., 2018; Bhattacharjee et al., 2019; Lamontagne-Proulx et al., 2019; Viodé et al., 2019). If a CSF sample is contaminated by blood, then α-syn levels are increased.

### Top-Down Approaches

Top-down proteomics analyze intact proteins by mass spectrometry and enables the study of the proteoform. This approach provides high information content on protein identification, differentiates between proteins and proteoforms, detects and characterizes post-translational modifications.

Intact protein analysis of α-syn from human postmortem brains identified 11 proteoforms (Kellie et al., 2014) including truncated forms confirmed from a Glu-C protease digestion protocol. The number of proteoforms identified in human postmortem brains was increased to 20 using both top-down and bottom-up approaches (Bhattacharjee et al., 2019) including modifications on the N-ter acetylation and truncations (on position 47, 57, 63, 64, 67, 70, 71, 79, 83, 86) and C-ter truncations (on position 55, 73, 94, 101, 103, 104, 119, 124), all described in Tables 1, 2. Intact α-syn proteoforms were also detected in appendix and brain from PD patients and controls (Killinger et al., 2018) including truncations such as C-ter truncations on position 114, 124 and 125 and also N-ter truncations on position 18 and 19. The detection of α-syn proteoforms in the appendix of both healthy controls and PD patients supports previously reported findings using immunohistochemistry techniques.

### Bottom-Up Approaches

In bottom-up proteomics, proteins undergo proteolytic digestion to generate short peptide fragments which are analyzed by tandem mass spectrometry (LC-MS/MS). This workflow has been applied to the analysis of α-syn in biofluids and target tissues (Table 4).

**TABLE 4:** Summary table of synuclein studies by mass spectrometry.

References	Matrix	Protein form	Sample preparation	Cohort	Synuclein peptides	Clinical levels
Yang et al., 2017	CSF (200 μl)	Total α-syn	PP and trypsin (and ASP-N) digestion	15 control, 15 PD *2	α-syn 13–21 and 81–96	Lower in PD vs control (correlates with diseases severity)
Oeckl et al., 2016	CSF (200) μl	Total α-syn, β-syn and γ-syn	Fractionation and trypsin (coupled to LysC) digestion	23 PD, 17 PDD, 10 LBD, 20 PSP, 10 CBS, 19 AD, 10 CJD and 37 control	α-syn (ac)-1–6, α-syn 1–6, 13–21, 24–32, 35–43, 46–58, 81–96; β-syn-46–58, 61–85 and γ-syn- 46–58, 81–96	α-syn, β-syn and γ-syn were significatively higher in AD and CJD but not PD vs control
Oeckl et al., 2020	CSF (200) μl and serum (950 μl)	Total β-syn	IP and trypsin (coupled to LysC) digestion	AD (Levine et al., 2017), control (Okochi et al., 2000), bvFTD (Jia et al., 1999), CJD (Flagmeier et al., 2016), ALS (Beyer et al., 2011). And 44 control, 40 AD	β-syn-46–58 and 61–85	Higher in AD and MCI vs control in both fluids
Bhattacharjee et al., 2019	Human brain tissue samples (cingulate cortex and occipital cortex)	Total α-syn, α-syn detergent soluble and insoluble fraction, α-syn truncations and acetylations	IP and trypsin and GluC digestion	PD (Oeckl et al., 2018) stage 5 or 6 and controls (Oeckl et al., 2018), stage 0 (Hoehn and Yahr score) *2	α-syn (ac)-1–6, 13–21, 24–32, 35–43, 46–58, 81–96 and 105–120	No significant differences level between soluble or insoluble fraction in PD patient vs control
Viodé et al., 2019	CSF (200 μl)	Total tau and α-syn	PP and trypsin coupled to GluC digestion	7 control, 10 DLB and 6 AD	α-syn 14–20, 36–43, 47–57, 62–80, 84–96, 106–114 and 132–140	All tau and synuclein peptides were significantly increased in CSF from AD compared to DLB and controls.
Kellie et al., 2014	Postmortem brain (frontal gyrus)	Total α-syn, α-syn detergent soluble and insoluble fraction, α-syn truncations and acetylations	IP and digestion with Glu-C	16 control, 10 PD and 6 AD	71–140; 68–140; 65–140; Ac 1–101; 39–140 Ac 1–119; Ac 1–122); Ac 1–135; 5–140; Ac 1–140	No significant differences level in PD patient vs control
Killinger et al., 2018	Human appendix and brain tissues (Substantia nigra)	Total α-syn and α-syn truncations	IP	appendix tissue: 8 control and 6 PD, brain tissues: 6 control and 6 PD	None	Patient with appendectomy have lower risk of PD and delayed the age of onset symptoms appearance

CSF is the most well studied biofluid for biomarker discovery in neurological diseases given its proximity to the central nervous system and the need for an informative clinical biomarker. As α-syn is a low abundance protein, LC-MS/MS methods have been developed to increase digestion efficiency (for example; IP, trypsin coupled to LysC digestion) (Oeckl et al., 2020), enhance specificity by considering specific signature peptides (7 α-syn peptides have been quantified, including the C-terminal peptide (132–140) for the analysis of tau and α-syn) (Viodé et al., 2019) and increase precision and robustness (using stable-isotope labeled proteins to account for variable total protein concentration between samples) (Oeckl et al., 2018).

Using two enzymes, Glu-C and trypsin for optimal sequence coverage of α-syn, Viodé et al. (2019) reported that CSF α-syn concentrations were significantly lower in DLB patients compared to AD or controls, and that tau and α-syn concentrations showed opposite trends in AD and DLB patients. Oeckl et al. (2020) applied immunoprecipitation-mass spectrometry (IP-MS) to AD patient samples and found increased concentrations of β-syn in CSF and blood compared to controls. β-Syn was also increased in patients with mild cognitive impairment, markedly increased in Creutzfeldt-Jakob disease but not behavioral variant frontotemporal dementia, dementia with Lewy bodies/Parkinson’s disease dementia, Parkinson’s disease, or Amyotrophic lateral sclerosis.

In brain tissue homogenate analysis, Bhattacharjee et al. (2019) identified higher concentrations of Ac-α-syn1–6, α-syn13–21, α-syn35–43, α-syn46–58, α-syn61–80, and α-syn81–96 in the Lewy body-enriched α-synuclein fraction from the PD cingulate region compared to controls (Lamontagne-Proulx et al., 2019).

Despite the extensive studies into α-syn proteoforms in CSF, plasma/serum, brain tissue, appendix and saliva (Guerreiro et al., 2012; Kellie et al., 2014; Oeckl et al., 2016; Killinger et al., 2018; Bhattacharjee et al., 2019; Mollenhauer et al., 2019) it appears that total synuclein concentrations are global markers of synaptic degeneration rather than specific clinical diagnostics for synucleinopathies. This may, in part, reflect methods which do not analyze the complete α-syn sequence particularly in the N-ter and/or C-ter region of the protein.

## Conclusion

Parkinson’s disease is a complex neurological disorder which is increasingly considered to be a group of neurological conditions and not a single disorder and for which there is no known cure. The complexity of PD results in several clinical challenges as the disease itself is highly heterogeneous with marked intra-individual and inter-individual variability. Clinical diagnosis of PD is based upon a combination of clinical assessments including history, physical examination with neuropsychological testing and imaging (dopamine transporter single-photon emission computed tomography scans). At the time of diagnosis, the neurodegenerative pathology is substantial and irreversible. For this reason, the identification of reliable, specific and robust biomarkers of PD would help improve diagnostic accuracy, provide tools to monitor disease progression and treatment response, and may also identify accurate patient stratification in clinical studies.

Clinical biomarker research in PD have suggested that the presynaptic protein, α-syn, may be a potential diagnostic and prognostic biomarker for PD and the related synucleinopathies given its association with PD pathogenesis (Kang et al., 2016; Mollenhauer et al., 2017, 2019; Yang et al., 2017). Extensive studies have identified t-α-syn, o-α-syn and pS129-α-syn as potential markers as they closely reflect pathophysiology of PD. Most studies have observed a decrease in t-α-syn and an increase in pS129-α-syn and o-α-syn in the CSF of PD patients (Kang et al., 2016; Yang et al., 2017; Levine et al., 2019; Mollenhauer et al., 2019; Chalatsa et al., 2020; Sorrentino and Giasson, 2020; Ganguly et al., 2021).

However, although potential PD biomarkers have been identified, the clinical utility of robust biomarkers is lacking due to small differences in the mean levels of α-syn species compared to controls and the high variability of single values. This variability may be attributed to clinical heterogeneity in the disease state of individual patients or in the methods used to detect α-syn species. The variability in antibody-based detection may contribute to different study outcomes particularly when different techniques and antibodies are used. Immunoassay methods are also limited by the availability of specific and available antibodies for the quantitative analysis of novel α-syn species biomarkers and by the need for standardization which has proved difficult. Conversely, as an antibody-free technique, mass spectrometry has the capability for identifying and quantifying a high number of protein targets in a single run with high sensitivity and accuracy. MS methods also deliver greater standardization and have greater flexibility in multiplexing [for example, MS can distinguish not only t-α-syn, o-α-syn and pS129-α-syn but also the truncated and acetylated form of the α-syn which tends to be higher in PD patient samples when compared to a control group (Bhattacharjee et al., 2019; McGlinchey et al., 2021)].

The challenge is to develop detection methods with the sensitivity and selectivity required for PD which can be applied to biomarker discovery and validation studies particularly as α-syn driven pathogenesis is likely to be multifactorial. Identifying robust disease markers will help not only our understanding of synuclein biochemistry in PD but the translation of such knowledge into future disease-modifying drugs for PD patients which is urgently needed.

## Author Contributions

All authors contributed to the design and implementation of the research and to the writing of the manuscript.

## Conflict of Interest

M-LP, NL, and SM were employed by the company Shimadzu Corporation. The remaining authors declare that the research was conducted in the absence of any commercial or financial relationships that could be construed as a potential conflict of interest.

## Publisher’s Note

All claims expressed in this article are solely those of the authors and do not necessarily represent those of their affiliated organizations, or those of the publisher, the editors and the reviewers. Any product that may be evaluated in this article, or claim that may be made by its manufacturer, is not guaranteed or endorsed by the publisher.

## Abbreviations

AB: antibody
AD: Alzheimer’s disease
CJD: Creutzfeldt Jacob disease
CSF: Cerebro-spinal fluid
DLB: Dementia of Lewy body
ICA: isotope-coded affinity tag
ICPL: isotope-coded protein labeling
IP: immunoprecipitation
LB: Lewy body
LC: liquid chromatography
MCI: mild cognitive impairment
MRM: multiple reaction monitoring
MS: mass spectrometry
MSA: multi system atrophy
NAC: non-amyloid component
PD: Parkinson’s disease
PDD: Parkinson’s disease dementia
PSP: progressive supranuclear palsy
PTM: post translational modification
pS87: phosphorylation on serine 87
pS129: phosphorylation on serine 129
PSAQ: protein stable absolute quantification
QrPREST: quantitative protein epitope signature tags
SIL: stable isotope labeling
SILAC: stable isotope labeling by amino acid in cell culture
SNc: subtantia nigra pars compacta
α-syn: alpha synuclein
o-α-syn: oligomer alpha synuclein
t-α-syn: total alpha synuclein
β-syn: beta synuclein
γ-syn: gamma synuclein
UPDRS: unified Parkinson’s disease rating scale
UPS: ubiquitin proteasome system
Vs: versus
WISIL: winged stable isotope labeled.
